# Attitudes of European psychiatrists on psychedelics: a qualitative study

**DOI:** 10.3389/fpsyt.2024.1411234

**Published:** 2024-05-24

**Authors:** Marija Franka Žuljević, Nando Breški, Mariano Kaliterna, Darko Hren

**Affiliations:** ^1^ Department of Medical Humanities, School of Medicine, University of Split, Split, Croatia; ^2^ Department of Psychology, Faculty of Humanities and Social Sciences, University of Split, Split, Croatia

**Keywords:** psychedelics, psychedelic therapy, psilocybin, MDMA, attitudes, psychiatrists

## Abstract

**Introduction and aim:**

It is important to understand how mental health practitioners view recent findings on psychedelic-assisted psychotherapy (PAP) as there is potential this treatment may be incorporated into clinical practice. The aim of our study was to explore how psychiatrists who are not involved in psychedelic research and who are located in the European region perceive psychedelics and PAP.

**Methods:**

We conducted online semi-structured interviews with 12 psychiatry specialists and psychiatry trainees from 8 European countries. Data were analyzed using a general inductive approach informed by codebook thematic analysis.

**Results:**

Based on the interviews, we developed four main themes and 14 sub-themes, including (1) Psychedelics hold potential, (2) Psychedelics are dangerous, (3) Future of psychedelics is uncertain, and (4) Psychiatry is ambivalent toward psychedelics.

**Discussion:**

Our respondents-psychiatrists acknowledged the potential of PAP but remained cautious and did not yet perceive its evidence base as robust enough. Education on psychedelics is lacking in medical and psychiatric training and should be improved to facilitate the involvement of mental health experts in decision-making on PAP.

## 1 Introduction

Psychedelics are hallucinogenic psychoactive substances with action at the serotonin 2A (5-HT2A) receptor (1). These substances have a controversial history of use that led to restrictive governmental policies in the 70s, after which the first wave of research on psychedelics came to a standstill (2). Recently, however, psychedelic-assisted psychotherapy (PAP) has experienced renewed research interest, and there are now implications that it may be effective in treating various forms of mental illness when co-administered with psychotherapy (3–5). The largest number of studies on psychedelic-assisted psychotherapy (PAP) involve 3,4-methylenedioxymethamphetamine (MDMA) and psilocybin, which have been designated by the U.S. Food and Drug Administration (FDA) as “breakthrough therapies” for posttraumatic stress disorder and treatment-resistant depression, respectively (6). So far, the United States (US) have been at the forefront of the modern era of psychedelic research in terms of clinical studies (7). Although the National Institutes of Health (NIH), the main public funding body for research in the US, was hesitant to award any research grants for PAP clinical trials in the past (8), Johns Hopkins Medicine received the first-ever NIH grant in 2021 to study the effect of psilocybin on tobacco addiction (9). Europe has also seen recent developments related to PAP; in early 2024, the European Union announced the allocation of a €6.5M research grant to explore the effectiveness of psilocybin for treating psychological distress in people with progressive incurable illnesses (10). These research efforts are taking place in a context where psychedelics, for the most part, remain illegal for recreational and, typically, for medical use as well (11). Although the prevalence of their use is still relatively low in Europe, this may soon change due to a rapid increase in media coverage of PAP and psychedelics in general (11). Overall, these research funding initiatives signal a potential upcoming increase in public discourse about PAP and its clinical and legal status in Europe, where mental health professionals such as psychiatrists will be likely candidates for discussions and recommendations about the topic of psychedelics in clinical practice.

In this rapidly evolving landscape of psychedelic therapies and legislation, it is vital to understand in-depth how mental health practitioners view psychedelics, as they are qualified to assess the implications of their use for the treatment of mental disorders, as well as potential future PAP providers. A 2023 systematic review of quantitative surveys of attitudes on psychedelics indicated that there is baseline support for further psychedelic research among mental health professionals coupled with curiosity and interest but that this group reported poor knowledge on psychedelics and PAP (12). Additionally, these surveys showed that mental health professionals, along with other stakeholders such as patients or members of the public, showed concern about the potential risks and side effects of psychedelics and PAP. Health professionals, specifically, were concerned with psychiatric and neurocognitive risks and emphasized that some populations may be more vulnerable to PAP-related risks (12). These concerns are further contextualized by the position statement from the American Psychiatric Association, issued in 2022, warning about the short- and long-term risks of psychedelics and emphasizing that they are not yet approved for medical use (13).

Given the psychedelics’ history and their present legal status worldwide, both psychedelics and PAP are topics that require a broad and complex discussion approach. In this context, qualitative research can be superior to quantitative surveys because it allows for in-depth exploration that can identify issues and capture nuances that quantitative methods might overlook. Qualitative research so far focused only on the perceptions of cancer health care workers and palliative care workers on psychedelics, with results showing some optimism but also concerns about trial design, dosing, real-world applicability, and the potential side effects of psychedelic therapies (14–16). Given the paucity of qualitative research involving participant groups such as psychologists or psychiatrists, we still don’t fully understand the nuances behind their views on psychedelics and PAP that are specific to their professions and the patients they see on a daily basis.

In this context, our study aimed to explore the perceptions of European psychiatrists on psychedelic-assisted psychotherapy using a qualitative methodological approach.

## 2 Methods

### 2.1 Study design and theoretical framework

We conducted this qualitative study using web-based interviews via scheduled video calls on the Microsoft Teams online meeting platform. We followed a realist pragmatic approach in this study as we aimed to focus on the practical implications of the psychiatrists’ perspectives toward PAP. As our methodological approach, we followed Braun and Clarke’s framework for conducting thematic analysis (17). However, our analysis remained at a descriptive level, rather than interpretive or reflexive. Thus our methodology is best described as codebook thematic analysis (17, 18).

### 2.2 Research question

Our topic of interest was psychedelic-assisted psychotherapy, seen through the perspective of psychiatrists and psychiatry trainees. We broke down our research question according to the SPIDER formulation (19):

(S)ample – Psychiatrists and psychiatry trainees working within the European region (based in any European country; not confined to the European Union only).
(P)henomenon of (I)nterest – Psychedelic-assisted psychotherapy
(D)esign – Interview study
(E)valuation – Perceived issues and implications for clinical practice, the design of future clinical trials, as well as policy
(R)esearch type – Qualitative

Our research objective was *to gain a deeper understanding of European psychiatrists’ perceptions related to psychedelic-assisted psychotherapy*. To obtain this objective, we formulated the following research questions:

1. How do European psychiatrists perceive psychedelics and PAP?
2. What do European psychiatrists see as facilitators or barriers to research on this topic, as well as the implementation of such therapies in a clinical setting?
3. What do European psychiatrists consider to be the implications of psychedelic research and/or the real-world implementation of PAP for their own practice and psychiatry in general?

### 2.3 Participant selection and recruitment

Our sample consisted of European psychiatrists or psychiatry trainees that were based within any European country. There were no formal exclusion criteria for participants. Participants were invited to the study via e-mail. We used a purposive sampling approach to obtain a heterogeneous sample that would best represent various practitioners in psychiatry who aren’t involved in psychedelic research. To reach eligible participants, we used different channels. One was through our personal and professional contacts. The second channel was from our other online study using an instrument to measure attitudes on psychedelics in European psychiatrists. In that study, we recruited psychiatry specialists and trainees by contacting professional psychiatrist organizations (all member organizations within the European Federation of Psychiatry Trainees and the European Psychiatric Associations), psychotherapeutic organizations, hospitals, and individual e-mails of corresponding authors on papers in psychiatry journals. Participants who filled out the survey were invited to leave their contact e-mail in case they were interested in participating in an interview for this study. Thus, this part of our strategy was a type of convenience opportunity sampling. Finally, individuals (via any of the sampling channels above) who agreed to the interview were asked to consider whether they had any colleagues who might be interested in being interviewed. In this way, we also used a snowballing sampling method.

Our sampling was guided by the demographic characteristics of the participants, which were continually reassessed with each new interview. We aimed to represent an approximately equal number of men and women, specialists and trainees, and to include participants from both Western, Central, and Eastern Europe. A previous survey we conducted indicated that there is a positive association between knowledge on psychedelics and attitudes on psychedelics (20). For this reason, we additionally chose to assess’ participants’ basic knowledge on psychedelics by a short objective test, already described in a previous publication (20). This test, combined with the authors’ assessment of their knowledge and level of informedness on psychedelics based on their interview, was used to reach a final judgment on their overall estimated knowledge on psychedelics, which was subjectively defined by the authors as low, moderate, or good. This was done to provide additional context to the participants’ statements.

We only chose to reach out to a smaller number of participants out of those who wanted to be contacted for an interview in our survey study (the second sampling channel), as most of them were younger trainees who were enthusiastic about psychedelics, so we didn’t want to overrepresent this population in our sample. Invitations via this sampling channel were sent sporadically since the data in the survey study was collected simultaneously with the interviews. Although our sampling approach may have reduced the final number of participants, we accepted this limitation in light of the considerations we made related to reflexivity (see *2.6 Research team and reflexivity).*

### 2.4 Data collection

Data were collected by conducting interviews and video and audio recording them during the ongoing conversation (the default recording option within Microsoft Teams). We chose interviews to collect our data because we wanted to gain deep insight in participants’ thinking and experiences related to the subject. Also, psychedelics and psychedelic-associated psychotherapy may be controversial topics for some participants so we preferred to meet our participant in a one-on-one setting rather than any form of group setting (e.g., focus groups). Interviews were conducted in Croatian (for Croatian participants) and English (for participants from all other countries). The recordings were used to transcribe the interviews subsequently. The verbatim interview transcriptions were de-identified and then further used for data analysis. We decided to stop data collection, i.e., conducting new interviews once the data saturation was achieved, according to the advice and parameters described by Hennink et al. (21). Hennink’s parameters were also considered when choosing new interview participants, along with the context of our study aim and the demographic diversity of the targeted sample. The interviews approximately lasted between 30 and 90 minutes. Upon transcription, all the interviews amounted to 114 pages (56914 words) of written text for the analysis.

### 2.5 Data analysis

Initially, we used pre-determined codes that closely followed our interview topic guide (e.g. “Psychedelics in research”, “Psychedelics in therapy”) in order to familiarize ourselves with the data and get its organized overview. After this step, we once again revisited each interview and coded anew using an inductive, bottom-up approach. For example, we created and used a code “Unrealistic expectations around psychedelics” for a corresponding participant quote: “*P11: There are risks of over-inflating the benefits, and that can leave some people sort of feeling quite helpless or feel quite disappointed if it doesn’t work the way that it works, like on a documentary for one person or in any story for somebody as well.*” Thus, the final themes were not set in advance but were derived based on interview data and developed during coding. However, we kept the codes, themes, and sub-themes at a semantic level rather than focusing on any latent meaning. We followed an iterative data analysis process, coding the data as new interviews were conducted. We constructed a preliminary codebook and thematic map after six interviews. This codebook was continually refined until saturation was reached. Consensus was reached on all authors’ interpretation and coding choices at multiple points throughout the iterative coding process and once again after the final themes and sub-themes were determined.

We used the NVivo (QSR International Pty Ltd., London, UK) qualitative data analysis software to manage the analysis. All other authors of the final publication who did not participate in the coding read all of the transcripts to verify that the final themes and sub-themes fairly represented the data set.

### 2.6 Research team and reflexivity

Marija Franka Žuljević (MFŽ), the study’s principal investigator, conducted all the interviews. MFŽ is a medical doctor employed as a teacher at the University of Split School of Medicine at the time of the study. She had received previous training in qualitative research and had previous experience in conducting focus groups and interviews. All participants were informed that MFŽ was conducting a PhD on the topic of psychedelics and psychedelic-assisted psychotherapy. Three participants knew of MFŽ through personal contacts but had no significant previous interaction before the interview.

Reflexivity was one of the chosen strategies for increasing the credibility of the study due to the controversial nature of the topic of psychedelics and psychedelic-assisted psychotherapy. Generally, the research team recognized their own positive biases toward psychedelics and PAP at the beginning of the study. In order to avoid overrepresenting themes that resonated with our own experiences, we made conscious choices to also include participants with more limited knowledge on psychedelics or with more negative or neutral attitudes on PAP. Although we had the opportunity to contact additional participants who were interested in the interview, we chose not to, since these were mainly younger individuals with more positive attitudes and higher knowledge on psychedelics, who were already represented among the interviewees (see *2.3 Participant selection and recruitment*).

To address these considerations, the author conducting the interviews and coding (MFŽ) kept a reflective diary throughout the data collection and analysis process to identify any potential personal biases that may influence communication with participants, as well as by identifying any personal attitudes held on psychedelic-assisted psychotherapy that could influence data analysis. This reflective diary also helped ensure a rich and balanced interpretation as the end-product of data analysis within the study. The senior co-author (DH) is a psychologist who teaches a course on qualitative methods. He was actively involved in the coding process, providing support and supervision throughout the data analysis process and creating thematic maps to ensure high methodological integrity and quality.

Interestingly, the research team as a whole identified that our own attitudes on psychedelics and PAP changed throughout the course of this study, as we were exposed to a plurality of perspectives and opinions. These reflexivity practices, together with the participants’ accounts, led us to a more cautious and balanced view of the future of psychedelics and PAP.

### 2.7 Ethical aspects

Ethical approval for the study was obtained from the Ethics Committee of the University of Split School of Medicine (document No. 2181–198-03–04-22–0007). All interviewees were presented with an informed consent letter with information about the study and what participation entails. They provided written informed consent by signing this document and sending it to the study authors in PDF form via e-mail.

Persons invited to participate in the study were free to decline without stating their reasons for doing so. They were informed that they could withdraw from the study at any point before their interview and up to one week after the date of the interview.

## 3 Results

**Figure 1:**
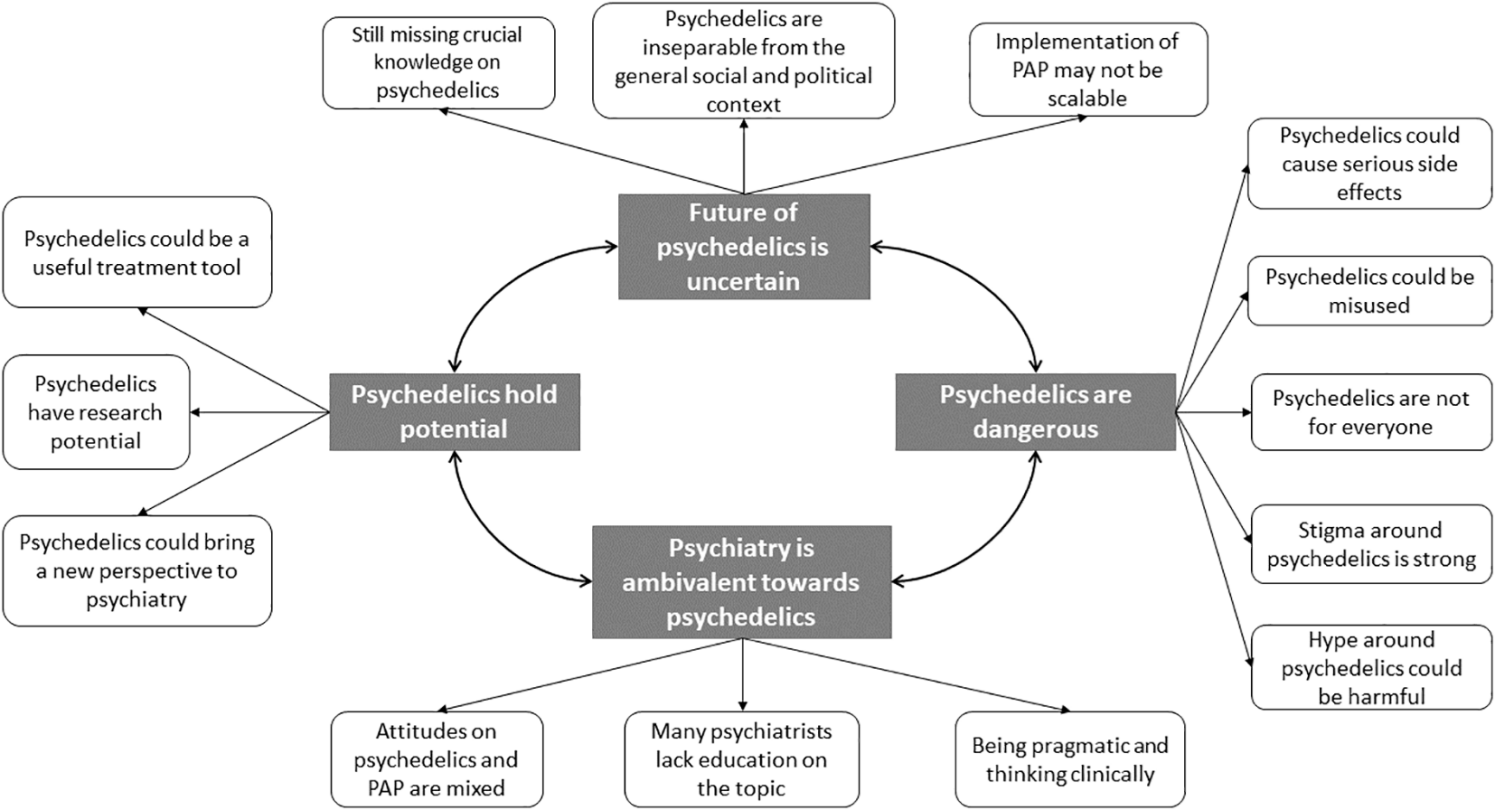
Thematic map of findings with relationships between themes and sub-themes.

We conducted 12 interviews with participants from 8 different European countries: Croatia, Poland, Sweden, Netherlands, Ireland, Italy, United Kingdom, and France. We included an equal number of men and women (n=6), predominantly psychiatry specialists (n=8), with a mean age of M=36.0 years (standard deviation [SD]=5.47). We defined four main themes and 14 sub-themes within the collected data (see **Figure 1**). Participant quotes are provided within each sub-theme, and the full demographic information for each participant from P01 to P12 is presented in **Table 1**.

**Table 1:** Demographic information of all 12 interview participants.

	Country	Gender and age	Education	Primary place of work	Primary treatment approach	Number of published articles	Estimated knowledge on psychedelics
P01	Croatia	Male, 30	Psychiatry trainee, psychotherapy trainee	Hospital	Biological	0	Low to moderate
P02	Croatia	Female, 43	Licensed psychotherapist, psychiatry specialist, PhD	Currently unemployed	Both biological and psychotherapeutic	13	Moderate
P03	Poland	Female, 34	Psychiatry trainee	Hospital	Biological	0	Good
P04	Poland	Male, 30	Psychiatry trainee, psychotherapy trainee	Hospital	Both biological and psychotherapeutic	10	Good
P05	Sweden	Male, 39	Psychiatry specialist, licensed psychotherapist, psychotherapy trainee	Outpatient clinic	Psychotherapeutic	5	Good
P06	Netherlands	Male, 40	Psychiatry specialist, PhD	University	Both biological and psychotherapeutic	70	Good
P07	Croatia	Female, 39	Psychiatry specialist	Hospital	Both biological and psychotherapeutic	10	Moderate to good
P08	Ireland	Female, 48	Psychiatry specialist	Hospital	Both biological and psychotherapeutic	1	Low to moderate
P09	Italy	Male, 31	Psychiatry specialist, PhD student	Hospital	Both biological and psychotherapeutic	17	Good
P10	Sweden	Female, 35	Psychiatry specialist, PhD	University	Both biological and psychotherapeutic	11	Good
P11	United Kingdom	Male, 34	Psychiatry trainee, psychotherapy trainee	Outpatient addictions center, inpatient addictions ward	Both biological and psychotherapeutic	0	Good
P12	France	Female, 36	Psychiatry specialist, PhD	University	Both biological and psychotherapeutic	18	Good

### 3.1 Psychedelics hold potential

The first theme encompassed all positive mentions of psychedelics and their worth or utility. It captured the statements of participants on positive attributes of psychedelics that make them carry the potential for treating patients, providing new insights into the mind or a new treatment model of psychiatry. Within this theme, we identified three sub-themes.

#### 3.1.1 Psychedelics could be a useful treatment tool

Participants highlighted many of the characteristics of psychedelics’ effects and the PAP model as potentially facilitating and speeding up the treatment process. Psychedelics were mentioned as a tool that could help access unconscious contents, “*get to the root of the problem*” (P11), and generally bring back the focus on patients and their insight and emotional processing:

*P03: I would say that [PAP] allows us to kind of explore our psyche or consciousness at different levels and different dimensions. So it kind of lets us find out more about the mind as kind of treating that individual as a whole, as a complex person, really kind of lets us go into the different areas of our mind. So in that sense, it helps us better understand the person, maybe better understand the symptoms that that person is having, better understand the causes like the why, which is always a very important question in psychiatry.*

Overall, this sub-theme highlighted that psychedelics and PAP have certain unique advantages that make them useful and give them strong potential as treatment methods.

#### 3.1.2 Psychedelics could bring a new perspective to psychiatry

Our interviewees saw the treatment potential psychedelics hold as potentially very attractive in the current context of psychiatry. The lack of innovations in psychiatry was considered as a basis upon which psychedelics and PAP offered a new and fresh perspective, holding a potential to reconcile differing and, at times, opposed treatment approaches in psychiatry.

Overall, participants expressed a lack of new treatment methods and innovation in psychiatry:

*P12: So because there is this lack of research in pharmaceutical industry, we are now stuck in a period where we don’t have new treatments to propose to patients. [When] you are a doctor, you have people that are really troubled and you can only propose SSRI (laughs) or older treatment. And then it’s electroconvulsive therapy – I mean, just painkilling for very sick people. And this is not enough, we need new treatments. We need to think out of the box of what we have. (…) And I think people go back to the interest in psychedelics because they are desperate and they say, “well, maybe we missed something at that time”.*

The new approach of using psychedelics as therapeutics was likewise mentioned as providing a new, non-conventional model of treatment that could bridge the existing gap between psychiatry and psychotherapy and bring back focus on psychotherapy in general, as a contrast to the existing predominance of pharmacotherapy:

*P10: [PAP], it’s a whole different kind of track. And I think that may be gaining some interest as a whole new, different way of thinking about psychotherapy, in a way.*

*P02: I am looking forward to new approaches to treatment, especially this integration of psychiatry and psychotherapy because, unfortunately, psychotherapy is not given enough space and more and more people are talking about the importance of integrating it.*

The need to improve outcomes for patients in psychiatry for new solutions was seen as a highly relevant reason for the ongoing consideration of psychedelics as a potential therapy and personally staying attentive for further research results.

#### 3.1.3 Psychedelics have research potential

Furthermore, our respondents described the research on psychedelics conducted so far as “*promising*” (P03), “*interesting*” (P04, P06), and “*exciting*” (P11).

Participants also expressed that psychedelics deserve further attention because further research in this field could help provide useful insight into how the human mind functions. Additionally, the involvement of respectable institutions in the field was reported to inspire interest and confidence:

*P12: I think it will lead us to a better understanding of what is a change in the human mind, but not on a biological point of view because it’s not so interesting, but also, in the dynamic way.*

*P05: When I hear that Johns Hopkins took on something [like this], that they started so many projects, well, I think there’s something interesting there. (…) It’s good that serious institutions are working on this.*

### 3.2 Psychedelics are dangerous

Although some participants highlighted the positive benefits and potentials of psychedelics, the idea that psychedelics are dangerous was very present and covered multiple aspects, from the side effects and perceived potential for abuse to the general stigma they carry as drugs and the way that the hype and enthusiasm around them can be harmful. This theme covered both the personal perceptions of the participants, their observations about what other individuals think, as well as participants’ impressions of the ongoing events in society and research. This was the “richest” theme and we identified five sub-themes within it.

#### 3.2.1 Psychedelics could cause serious side effects

Psychedelics as substances were perceived by the participants as having numerous potential side effects, especially the risk of abuse/dependence, and of patients manipulating to gain access to the substances:

*P02: I believe that that feeling [on psychedelics], it can be something that people who are deep in the throes of addiction might want to return to.*

*P01: So there is a certain personality structure in people who tend to simulate a certain psychiatric condition in order to get what they want. If we open the way for them so that, instead of going to a dealer, paying for psilocybin, LSD, whatever – we enable them to get it at the expense of the state because they get a diagnosis – those things will definitely happen.*

However, not all participants agreed with the view of psychedelics as addictive:

*P09: I read and I am quite confident that the dependence that has been raised as a reason to not to do psychedelic assisted psychotherapy is not a real threat.*

*P04: And I suppose maybe a few sessions [for substance users] with psychedelics like psilocybin, DMT, LSD would change their view and maybe it would be easier for them to not to come back to drugs once and for all.*

Participants also said they were worried that using psychedelics within PAP could cause traumatic experiences the patient would not be able to handle such as a “*too heavy fragmentation or ego dissolution*” (P09) and that psychedelics could exacerbate existing or underlying psychotic or manic phenomena:

*P11: I think that there is reasonable evidence to show that if someone has a family history or a direct history of potentially psychosis or manic phases, then they can trigger that response in people.*

Overall, participants concluded that the risks for patient decompensation, if present, could be decreased by taking increased safety measures, such as a “*good medical interview*” (P04) or a “*general history taking*” (P03) to screen patients, “*a safe setting*” (P07), or “*good medical ethics committees that are checking the studies*” (P06).

#### 3.2.2 Psychedelics could be misused

Besides the immediate side effects of psychedelics, participants reported concern about the general misuse of psychedelics in ways that were not intended by PAP protocols. They highlighted a risk that therapists providing psychedelics could abuse their role and power, especially if not trained or supervised properly:

*P03: So, again, who is administering this session to us? Can we trust them and will it be just used for our own good?*

*P06: So you get a lot of obscurity with maybe psychologists that are going to do it with psychedelics, but not on recipe, but just in private practice. I think that will be a danger.*

Even when not seen as causing dependence, psychedelics were also perceived as having the potential for substance abuse. Some participants were concerned that they could be a means of escapism or that patients could start relying on them too much instead of doing the therapeutic work themselves:

*P04: In these patients, psychedelics can be harmful in a way that they just want to get high and then they don’t want to be sober because of some reason.*

*P03: So maybe [they will] start really relying just on this substance rather than looking at the therapy as a whole.*

Participants offered some solutions to these risks highlighting that therapists could be “*in supervision groups*” (P11) and that they should be “*checked in some way*” (P06). A way patients could be protected from therapists is that *“[psychedelic] séances could be filmed*” (P02) and that PAP should stay “*within the walls of a hospital*” (P06). Likewise, patients should “*understand why they’re doing [PAP]*” (P03) and that “*one should define what the goal of the therapy is*” (P05).

#### 3.2.3 Psychedelics are not for everyone

The risks and side effects of psychedelics also translated into a general view that psychedelics are not for everyone. This could be seen through accounts of participants when they talked about how it’s difficult to choose which patients would be eligible for PAP, both psychologically and physically:

*P11: Often people [with alcohol problems] are a little bit more frail, so I guess we need to be making sure that people are physically and physically safe to get it.*

*P11: At the moment, things like personal or family history of psychosis or personal family history of bipolar tend to be ruled out, although there’s a strong movement of people in the bipolar community to say that that’s actually being a little overly cautious.*

Additionally, since psychedelics can lead to changes in personal beliefs and views, this was seen as something that some individuals may not want or need:

*P04: [Psychedelics] can change the more important domains. Like, for example, how someone is thinking about religion, about political views, because the feeling after psychedelics, it’s this feeling that you are connected with the world. So it can change you as a person as a whole. (…) Maybe the patient would be grateful for this change, but we have to be aware that it is possible and maybe not everybody would like to change his point of view in some other domains.*

Some participants also reported an impression that psychedelics and PAP were not interesting to them because, for now, they do not see them as having the potential to treat patient groups they are mostly seeing, such as substance users (P07) and geriatric patients (P08).

#### 3.2.4 The stigma around psychedelics is strong

Psychedelics were described as “*controversial*” (P07), carrying a “*social stigma*” (P01), having a “*bad reputation*” (P10), and people having “*prejudice*” against them (P06).

In particular, the message “all drugs are bad” was mentioned as key to their stigmatized position in society:

*P04: Drugs are treated as a whole. And it doesn’t matter if these are psychedelics or opioid stimulants.*

This stigma was described as confusing when combined with the new idea that psychedelics as drugs could be used for treatment:

*P08: There’s the risk of mixed messages when we tell the public about, you know, “don’t use drugs” and yet we use drugs in a clinical setting.*

*P07: It would be very unusual for me [to do PAP], honestly, I can’t even imagine. So for us [in addiction psychiatry] patients - the prerequisite for coming for treatment is that they are sober, that they are clean. You can’t do psychotherapy with him if he’s spazzing out, right? So it’s a very unusual concept, actually.*

The stigma was not only related to drugs but also to psychedelics’ cultural and historical notoriety, most often mentioned concerning their role in the counterculture movement of the 60s, the secret government experiments, and the New Age spirituality movement:

*P01: For example, we hear quite often, from the hippies of the 60s onwards … we heard that, when it comes to psychedelics, they open the third eye, they act on, I don’t know, the chakras in the body…*

*P04: There are many [CIA programs] for which it’s supposed that they were conducted, but MK-ULTRA was documented and it is possible that they can be also used for brainwashing.*

The media was also described as a risk factor in further propagating the public stigmatization of psychedelics through the generation of negatively slanted and sensationalist news:

*P04: I suppose if it happens that we will have some psychedelic-assisted psychotherapy, there will be some stories about people who went to therapy or, I don’t know, got the psychedelics and wanted to make their own therapy but something bad happened to them, that they went to the hospital.*

*P06: Because if you have one big case with a suicidal patient, for example, that is on psychedelics, then people will be - the media and the overall sentiment against psychedelics would be big.*

Participants perceived the contrast between the potential and stigma as an issue that needs to be resolved, e.g., to “*solve the dichotomy [between drugs and medical use]*” (P05), and reduce the “*mixed messaging*” (P08). One solution offered to disassociate psychedelics from their stigma was “*rebranding (…) [by] changing the name so that it wasn’t associated with the street drug*” (P08).

#### 3.2.5 The hype around psychedelics could be harmful

Moving beyond the negative image of psychedelics from the past, participants also focused on serious concerns about the hype present around the current wave of psychedelic research, which generates unrealistic expectations of psychedelics as a “cure-all” among researchers, physicians, and especially for patients:

*P11: The media hype is being overly positive and there’s been a lot of headlines out of, not all that many trials, or not larger trials. So I think that there’s maybe a little bit of overhype and that can lead people to think that these are a miracle cure or like a golden pill that is going to fix everything.*

*P12: A lot of patients, they say they see the name of the drugs in the journal. They want the treatment in the hospital. Even if we say, no, you are not a good candidate.*

Furthermore, the media was also seen as playing a role in the hype and exerting pressure on researchers:

*P12: The media put pressure on the researchers and doctors to say that it is interesting and important (…) Yes, the journalists come and say, “OK, do you think the psychedelic is the new therapy?” And they say, “Oh yes, of course.” But they don’t have this public health way of seeing the thing. I mean, when you say this in the most read journal of France, what are you doing now?*

Besides the responsibility when speaking about psychedelics publicly (P12), some of the solutions participants offered to the risks carried by the hype were to “*stay humble (…) [and] always aim for more research*” (P06), “*always be a bit cautious*” (P10), and “*be very, very transparent in research*” (P09).

### 3.3 The future of psychedelics is uncertain

The third theme revolved around the general sentiment that it was unclear whether psychedelics and PAP would ultimately be successful in reaching clinical practice. This theme described general unknowns that still exist about psychedelics and which relate to research findings, social and political factors, and perceived factors influencing real-life implementation attempts. Overall, we identified three sub-themes.

#### 3.3.1 Still missing crucial knowledge on psychedelics

Participants said that the current evidence about psychedelics and PAP was still insufficient in their eyes. They especially emphasized the need for more extensive studies, primarily randomized controlled trials:

*P12: I need to see [with] my eyes first and then bigger data trials. Well done, multicentered, with good outcomes. I want to see effect on suicide. I want to see effect on going back to work, going out of the hospital, for instance, on suicidal ideation. I want to see this data. (…) For me, this is only pilot studies, but they are sold in the paper as phase three or phase four trials. This is bad science. It’s too early, too few - samples are ridiculous.*

*P02: There is already something, but it’s mostly still very small numbers, at least what I’ve read, is still relatively small numbers of subjects so there should be a certain number of positive results. Preferably compared to conventional treatments.*

Participants also expressed the idea that certain critical questions about psychedelics and PAP were still unanswered, making any speculation about the future more difficult. Some of these unknowns were reported as increasing the perceived risk of PAP:

*P03: I would worry about what happens if one, kind of, continuously over their life goes for these sessions. What kind of effect will it have then on our psyche?*

*P02: It seems to me that there are still no clear guidelines regarding dosage, frequency, treatment. (…) A lot of a kind of … preparatory work [is needed] before it can be applied on a wider scale.*

Participants also mentioned methodological issues with psychedelic trials that may be confounding accurate results. One such limitation that was brought up was a lack of standardization of psychotherapeutic techniques used within PAP, where an individual therapist’s style may introduce bias into the results on PAP’s effectiveness:

*P09: I think that there is not yet a standardized training, for example, for assisted psychotherapy and those in the past when it was tested, it was more up to the clinician, up to the therapist.*

*P06: I think it also is very dependent on the therapist if he knows what he or she knows what to do with these types of patients, then you have more chances that it’s actually effective.*

Another participant raised the issue of which outcomes are measured in clinical trials, and whether the chosen outcome measures are appropriate for an intervention like PAP, as they were originally developed to assess the effectiveness of different groups of antidepressants:

*P12: The FDA wants the Hamilton [scale] and the MADRS [scale] to be measured for market access, for the drug to go to the markets to be authorized [for treatment-resistant depression]. (…) These outcomes were tailored to show efficacy of tricyclic and SSRI. So Hamilton for tricyclics and MADRS for SSRI. So it’s just, like, impossible to show something [in the case of PAP], when you try something else.*

Finally, another question that was seen as relevant was that perhaps the psychotherapy segment of PAP may not be necessary and that the intervention may change its format in the future:

*P06: And I also think that the next step would also be that you don’t do psychotherapy anymore, you just use psychedelics and see whether that will help you as well. So that is another development that’s probably going to start.*

Some practical solutions to address these unanswered questions were to “*include patients when designing research*” (P09) and “*collect exploratory data with qualitative interviews [with patients]*” (P12).

#### 3.3.2 Psychedelics are inseparable from the general social and political context

Psychedelics were described as an issue deeply embedded in a social context. Participants said that any implementation of PAP was also inseparable from questions about the legal status of psychedelics. Likewise, research involving psychedelics was seen as potentially limited as long as psychedelics remained controlled substances in most countries worldwide:

*P09: I think [legalization and research] are connected, and I would agree with the liberalization and maybe also … legalization because of two reasons. The first reason is that we would be more free to investigate the substances and the second reason is that the people would take [them] for recreational use anyway, so it’s better to provide them with control and safe substance rather than a dirty or mixed compound that may be dangerous.*

Along with this, participants emphasized that financial interests could influence the future of PAP. They stated that psychedelics and PAP have the potential to be commercialized and subject to market-driven interests, but also that, if this area got more funding, this could be more interesting and relevant for them:

*P10: I know in the US a lot of, for example, IT companies have been pouring money into psychedelic research, and that also helps. I mean, where there is money that’s also going to be increased research.*

*P11: [My personal involvement] largely depends on funding, really. If it’s something that is likely to be funded, if it’s something that the government will fund, or even mental health charities or bodies are likely to get interested in funding. Then I think that I’ll be a lot more likely to work on it.*

Finally, psychedelics were described as connected to the current historical moment and zeitgeist and that their future would both influence and be influenced by ongoing changes in society and psychiatry:

*P09: I listened to some interviews of people that had a, for example, psychedelic trip or a psychedelic treatment, and they suggest also and they report increased connection with nature. So in the era of ambientalism, ecologism and so on it may be useful also for that, from a social perspective rather than a therapy setting.*

*P03: [Where psychedelic research will go] is kind of something that we don’t know much about, and when it comes to our mind, a very vast subject, I would kind of be a little bit hesitant as to what dimensions are we tapping into and what are we discovering, and are we ready to discover these different dimensions?*

This theme generally tied in with the sentiment expressed earlier that psychedelics are controversial, so some participants compared the situation with psychedelics with issues around cannabis legalization (P04, P09).

#### 3.3.3 Implementation of PAP may not be scalable

Participants expressed that the real-world clinical implementation of the PAP model may not be scalable. Primary, they stated that this is due to high staffing, administrative, time, and resource requirements:

*P10: And also, one thing when it comes to PTSD and psychotherapy, as I understand it, the psychedelic is like an individual therapist and patient treatment. That’s also not very staff-efficient. (…) And also, you need to have the administration of the psychedelic and the patient needs to be a certain time in hospital, as I understand. And then for observation afterwards, maybe not for a long time, but it’s a whole different way of setting up the care that I think is going to be logistically [difficult].*

*P12: This is very expensive, so it will [have to] be done in very specific departments as the one where I work, where we have a lot of money and you can pay psychologists to do this session. But basically, I don’t think we have the means to pay so much psychotherapy. So, it will be … I don’t think the cost effectiveness of this therapy, that it’s worth it.*

There was also a sentiment that such a complex treatment is not compatible with the current infrastructure, which is, in many places, already heavily overburdened:

*P10: But at least in Sweden, in my clinic, I mean, there’s already a lack of therapists for regular, more established PTSD treatments. For example, exposure, CBT. There’s currently a two-year waiting list just to get to specialist psychiatry, you know, CBT for trauma here. (…) If we can’t even provide the regular basic PTSD treatment [with] psychotherapy, I don’t know if we have the resources to [include] additional treatments under investigation.*

*P12: But in reality, we don’t have a nurse in the hospital. So one third of the beds are closed because we don’t have enough [staff]. So let’s talk about psychedelic psychotherapy, it’s impossible to do.*

Additionally, some participants were confident that the current model of PAP, with its high resource requirements, may be costly and only be available for well-off patients:

*P09: I have the fear that it will become something that only rich people may have access to, because psychotherapy is already very expensive and a new substances used as medication usually cost a lot of money at the beginning of the trading.*

### 3.4 Psychiatry is ambivalent toward psychedelics

Psychedelics were described as a topic that creates a lot of divided opinions and debates within the field of psychiatry. The “50/50” split was not only expressed in terms of attitudes but also significant discrepancies among individuals in the level of interest and knowledge on psychedelics and PAP. This theme referred to both opinions held by participants and those they used to describe as their colleagues or, more generally, wider groups of psychiatrists.

Although our participants saw opinions on psychedelics as mixed and likely to stay that way, many participants expressed that awareness about them is rising, e.g., *“[psychedelics] are starting to be discussed and talked about” (P08)*, *“there’s a growing demand for [education on psychedelics]”* (P11), and *“[I] recently attended a lecture on psychedelics and PTSD”* (P10). Overall, we identified three sub-themes within this theme.

#### 3.4.1 Attitudes on psychedelics and PAP are mixed

When asked about the potential introduction of PAP in real-life clinical practice, the participants generally expected the possible reaction of the psychiatric community to be “*ambivalent*” (P10), “*split*” (P09), or “*divided*” (P02, P03).

Participants gave two explanations on why their psychiatrist colleagues or the psychiatric community as a whole may be opposed to psychedelics. Firstly, they mentioned a fear of psychedelics’ side effects based on a broader and more general fear of substance use-related harms:

*P11: People in general psychiatry and adult inpatient wards seem to be a little bit more cautious, and I think that’s probably because they work in inpatient units, they said they often see when drugs go wrong, when people who probably shouldn’t be taking psychedelics take them and end up in hospital.*

Participants also saw a general resistance to novelty and change as a source of disinterest or opposition to psychedelics and PAP.

*P04: I think in general, most of psychiatrists would disapprove it, but they also don’t use the newest drugs, which seem better or better tolerated. They don’t read, they don’t learn, they just do. They just treat patients like they did 20 or 15 years ago.*

New interventions such as PAP, in particular, were mentioned as connected with fears and a certain resistance in prescribing new substances:

*P06: People are scared of prescribing new stuff, so that’s also the reason why it really takes care before it really gets implemented in a country.*

*P08: And where that, I suppose, the reason I talked about the past use is that there have been novel treatments in psychiatry, all going through the years that the last hundred years and some have not been successful. (…) So people are slightly wary, I think, of novel treatments until they’re seen to be safe and effective.*

Some psychiatrists were described as more open than others, especially those who were younger or already generally more open to novelty:

*P03: Some people who are maybe very like in favor of rapidly evolving treatments and kind of being on top of everything, up to date, maybe they would be for it.*

*P10: I think it is very different even now, just for the past five years, I see a shift in attitudes, especially with the younger generation.*

#### 3.4.2 Many psychiatrists lack education on the topic

Knowledge on psychedelics and PAP was a topic with significant division present in psychiatry, where mostly personally motivated individuals are reading about new research developments. Education on psychedelics was often described as lacking overall within one’s professional training, although some participants reported increasing awareness and discussion of the topic within the field. However, some participants said their colleagues could profit from more education when considering the topic:

*P03: It’s not something that is covered in depth, obviously, in medical school, and it’s not something that is the main area of focus in our residency training.*

*P06: Yeah, I think [the knowledge] is very basic still. It’s not very well developed yet. So training will take time before it’s actually there.*

A significant number of participants also expressed the personal view that they didn’t often encounter psychedelic users in their practice nor receive sufficient education on psychedelics during their medical school or psychiatry training. Any knowledge on the subject was described as left up to personal interest and initiative:

*P07: I don’t even remember that they were mentioned, maybe they were mentioned … well, LSD was the only one that was mentioned during my studies, and during my residency … I was in addiction psychiatry for a long time, but there were no such patients, the education was not somehow formalized then. So formally, no. It was left to me, right?*

*P11: There was a mention that I mean, I think if it was mention of LSD or magic mushrooms, it would have been called by our teacher as negative and [that] these are things that can make people mad, make people psychotic and end up in hospital.*

Some solutions to this lack of knowledge that participants suggested were “*continuing professional education program*s” (P11), “*discussing it on most [psychiatry] conferences*” (P04), “*have psychiatrists witness these sessions*” (P03). Likewise, the lack of knowledge and education was connected by participants with the fundamental skepticism and resistance many psychiatrists feel toward psychedelics.

#### 3.4.3 Being pragmatic and thinking clinically

Finally, participants expressed a pragmatic viewpoint, stating that, in the end, their primary focus is on their patients and clinical outcomes. This view encompassed that if they were presented with adequate evidence and backed by the field, they would be open to applying it in treatment. There was also a consideration that if professional organizations would give a favorable judgment of PAP, this kind of consensus would make it more acceptable to them. The distinction of thinking like a clinician rather than involving their personal attitudes was a significant difference emphasized within this view:

*P12: So I think that there is this feeling in the youth that are interested in psychedelics, but for me, as a doctor, it’s not a question of psychedelics or not. It is: do you have a new tool to help my patients or not?*

*P10: I think also what we’re interested in is “How this is going to change my current work clinically?”.*

*P08: And you know, most psychiatrists are pragmatic people in the end, and if they find a medication that is evidence-based, safe and effective, they will use it.*

Overall, participants, despite naming significant cautions toward psychedelics for the most part, were generally open to new developments and a change of attitudes or paradigm that could follow in their field.

## 4 Discussion

Through interviews with a sample of European psychiatrists, we found that they saw psychedelics through an ambivalent lens, with both caution and enthusiasm. They talked about psychedelics in terms of their potential as possibly beneficial treatment approach that could bring a new perspective and theoretical insights to psychiatry, which is in need of innovations. Here, PAP was seen as a reconciliation between pharmacotherapy and psychotherapy. At the same time, participants expressed caution about many different risks of psychedelics, including serious side effects like substance dependence and abuse, risks of psychotic and manic decompensation, and physical harm to frail patients. In general, they did not consider PAP suitable for everyone and talked about its risk of misuse for non-treatment-related goals by both patients and therapists. Psychedelics’ historical and drug-related stigma was contrasted by the ongoing hype around their potential, something that could be equally as harmful by giving patients and the public unrealistic expectations. Participants saw the future of psychedelics as uncertain, in light of missing answers to key methodological questions, and due to the high resource requirements of PAP. PAP was also seen as something the psychiatric community had mixed feelings about, which were amplified by a general lack of knowledge and systemic education on psychedelics. However, many of our participants expressed that, if presented with enough evidence and support from their profession, they and their colleagues would be likely to apply PAP.

Psychedelics and PAP are currently emerging topics without a large body of modern-era clinical evidence, leading to many unknowns and uncertainties as to what the future will bring to this line of research. Thus, the discussion around them is highly nuanced and controversial (7, 22, 23). Therefore, the main strength of this study is that we used qualitative methodology appropriate for the complexity of the topic and that we included participants with diverse personal and professional characteristics. A limitation to point out is that we included 12 participants, which may still give only a limited perspective on psychedelics and PAP. The representability of our findings for the whole population of European psychiatrists cannot be ascertained, as it is possible, if not probable, that some essential voices are missing. However, the aim of this study was not to give an overarching and final overview of the attitudes of all European psychiatrists but to explore and provide an overview of some of the central issues in the discussion around psychedelics and PAP. Our findings can, therefore, be considered a preliminary foundation upon which future qualitative studies could build upon, targeting more specific expert or psychiatrist sub-groups, such as individuals working with treatment-resistant patients or substance users. Finally, a note of caution is that future studies may consider using terms other than PAP when designing their study and interview guide. As psychedelic treatments are developing rapidly, psychotherapy may not always be the adjunct method following drug administration, warranting the use of different terminology.

Overall, our themes and sub-themes seemed consistent with insight from surveys conducted with psychiatrists so far. Our participants’ accounts were concordant with the general openness to using psychedelics in practice that the previous surveys identified (24–26). Similar to this, the caution toward possible risks and side effects of psychedelics was also described among participants in our study (25, 27). This comparison shows various nuances in psychiatrists’ attitudes that survey-based studies could not capture. Although developing dependence is a significant risk of psychedelic use (28–31), our participants highlighted concerns about substance abuse motivated by effects experienced on the psychological level, e.g., looking for easy solutions and escapism. The idea expressed by participants that patients could continue to use psychedelics after receiving them within PAP is supported by the findings that more side effects of psychedelics are present in unregulated and unsupervised use (31, 32). Participants’ concerns about psychosis and other forms of decompensation upon the use of psychedelics are partially supported by the lack of definite information on side effects such as psychosis. However, the new era of clinical trials on psychedelics has minimized harm to participants by using strict safety and screening protocols (28, 33). Finally, our finding of younger individuals being more personally engaged and open to PAP follows previous quantitative observations (20, 25, 34). Interestingly, younger participants in our study were the ones who offered many of the potential solutions and ideas in response to the negative aspects of PAP and psychedelics.

Our participants wanted more robust and convincing evidence on PAP, especially regarding bigger sample sizes and comparisons of PAP to standard treatments. They reported themselves and considered their colleagues to think about the issue of PAP pragmatically and in terms of what these new findings bring to their patients. With that in mind, an essential suggestion would be to communicate evidence and new findings to psychiatrists by focusing on clinically relevant information, such as patient-centered outcomes. Our participants also gave many suggestions for further research, such as including patients in study designs. Another important word of caution expressed by participants in our study was the discrepancy between the stigma of psychedelics as illegal substances and their newly emerging image as medical treatments. This discrepancy is something the psychedelic research movement should address, especially in communicating evidence to the public. Our participants mentioned negative examples of media using researchers to generate additional positive hype on psychedelics and PAP. These occurrences are a significant consideration as well, highlighting the need for responsibility and accountability from the psychedelic research community. Similarly, in the case of fentanyl use in North America, media sensationalism and misinformation may actually have a counterproductive effect by encouraging hyper-punitive laws and further stigmatization of substance use (35). In the case of both positive and negative media-generated hype, therefore, caution and an evidence-based approach is needed. The public’s need for psychedelic researchers’ trustworthiness was also demonstrated in a survey that showed that patients would view psychedelic researchers more favorably if they didn’t personally use psychedelics or, if they did use them, that this would be publicly and transparently disclosed (36).

Overall, our findings underscore the importance of increased personal transparency among psychedelic researchers, following previous similar comments about a positive expectancy bias among researchers conducting clinical trials with psychedelics (37). PAP appears to be a topic where non-medical discussion points and factors have the potential to complicate discussions around their medical use. Representing multiple perspectives on psychedelic conferences could alleviate this issue and bring the focus back to the evidence instead of merely encouraging further enthusiasm. Another criticism of the current psychedelic research is using terms such as “consciousness” vaguely and including religious icons in the psychedelic therapy process (38). If one aims to decrease the present cultural stigma toward psychedelics, also visible in our participants’ accounts, such a criticism is likely to be valid. It should be seriously considered and applied by using well-grounded terms when speaking of concepts related to psychedelics or consciousness. In addition to this, this study once again demonstrated the importance of educating professionals such as psychiatrists on psychedelics, as there does not seem to be enough knowledge in the field to follow the pace of research developments related to PAP. The perspectives of our participants highlighted the fact that psychedelics are missing in the curriculum of either medical studies or psychiatry training. Initiatives such as including educational packages on psychedelics at conferences or continuing professional development courses are an exciting consideration to improve the knowledge in the field.

## Data availability statement

The raw data supporting the conclusions of this article will be made available by the authors, without undue reservation.

## Ethics statement

The studies involving humans were approved by Ethics Committee of the University of Split School of Medicine. The studies were conducted in accordance with the local legislation and institutional requirements. The participants provided their written informed consent to participate in this study.

## Author contributions

MFŽ: Conceptualization, Formal analysis, Investigation, Methodology, Software, Writing – original draft, Writing – review & editing. NB: Writing – original draft, Writing – review & editing, Investigation. MK: Investigation, Writing – original draft, Writing – review & editing. DH: Conceptualization, Investigation, Methodology, Writing – original draft, Writing – review & editing.
